# Mechanical Reinforcement of Paper Biocomposites Using Filamentous Cyanobacteria

**DOI:** 10.1021/acssuschemeng.5c02889

**Published:** 2025-10-01

**Authors:** Sergio Serrano-Blanco, Priscila Melo, Adam P. Harvey, Sharon B. Velasquez-Orta

**Affiliations:** † School of Engineering, Merz Court, 5994 Newcastle University, Newcastle upon Tyne, NE1 7RU, U.K.

**Keywords:** Microalgae, cellulose, biorefinery, sustainable materials, pulp, biomass

## Abstract

Rising concerns over plastic packaging and the growing demand for e-commerce have increased paper-based materials production, thereby intensifying environmental impacts of the paper industry, one of the most polluting industries worldwide. The need to source nonwood fibers to alleviate environmental pressure has brought microalgae into the spotlight as a sustainable and renewable resource. For the first time, this study proposes and evaluates replacing cellulose fibers in paper-like biocomposites with the cyanobacterium *Leptolyngbya* sp. SB090721. The effect of using cyanobacteria as a cellulose replacement (0%, 3%, 30% w/w) was evaluated. Addition of cyanobacterial biomass (3–30%) maintained or enhanced tensile properties. The standard biocomposite showed the highest tensile strength (4.8 kN·m^–1^) and tensile energy absorption (195.63 J·m^–2^). Both the standard and high biomass composites showed enhanced elasticity moduli of 997.1 and 903.4 MPa. Significant structural differences were observed on the SEM micrographs, with the high biomass specimen displaying a distinct structure, attributed to its elevated cyanobacterial content. In conclusion, the study confirmed the feasibility of using unprocessed cyanobacterial biomass as a nonwood fiber source for paper and paperboard materials. This reduces the amount of cellulose used in the paper industry, offering new properties and production routes that could potentially be more sustainable.

## Graphical abstract

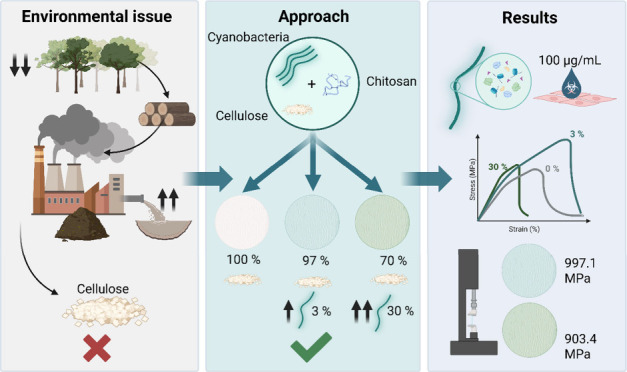

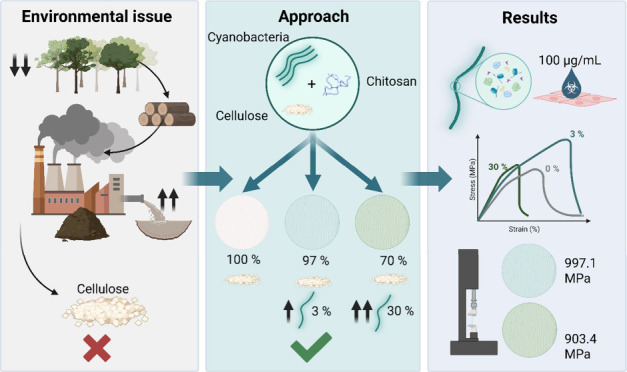

## 1 Introduction

Wood pulp production has increased 15.5% since the beginning of this century, and by 2032, paper consumption will reach 476 million metric tonnes.^1,2^ The growing demand for e-commerce, coupled with rising concerns about plastic packaging pollution, has increased paper-based packaging consumption. Consequently, the paper and pulp industry, the sixth most polluting industry, is generating substantial amounts of solid, gaseous, and liquid waste.^3,4^ The global demand for sustainable and ecofriendly materials has driven research into valorizing alternative biomass sources for various industrial applications, including paper production, biocomposites, and cellulose extraction. Thus, finding renewable nonwood fibers has become a priority to reduce environmental pressure.

Microalgae and cyanobacteria are sustainable biological resources that can fix and maintain CO_2_ while producing valuable biomass for industrial applications.^5^ Furthermore, microalgae present 10 times higher photosynthetic efficiency and 10–50 times higher CO_2_ fixation rates than terrestrial plants.^6,7^ Therefore, microalgae and cyanobacteria are a renewable feedstock that can contribute to meeting the Sustainable Development Goals (SDGs) and promote the circular economy.^8^ Microalgal biomaterials are a growing research area that comprises bioplastics,^9,10^ bioinks,^11^ biosilica,^12^ biocoatings,^13^ biofilters,^14^ biotextiles,^15^ biocomposites^16^ and building biomaterials.^17^

In pulp and papermaking, adding algae can contribute toward a more sustainable sector, as algae do not compete for arable land. The incorporation of microalgal biomass into biocomposite formulations can provide new functionalities to these materials. Additionally, residual microalgal biomass can be obtained as a byproduct of other processes, making it an interesting source for producing biodegradable biocomposites at lower costs.^18^ Biocomposites are safe materials, are easy to process, are light weight in nature materials, and have a reduced cost.^19^ Moreover, their environmental impact is also lower due to a reduced dependence on nonrenewable energy, pollutant emissions, and greenhouse gas contributions.^20^ Therefore, combining biocomposites with microalgae is a smart, sustainable strategy that can replace wood-derived materials in various industries (Table 1).

**Table 1:** Recent Microalgal and Cyanobacterial Biocomposites and Their Mechanical Properties^a^

**Biocomposite Material**	**Microalga Added**	**Microalgal Pretreatment**	**Outcome**	**Reference**
EVA	*Chlorella* sp.	Heat spray-drying, ball milling	60% microalgae content led to 61.02% tensile strength of pure EVA	^21^
PLA	*Arthrospira platensis*	Sonication	= Young’s modulus	^22^
			↑ 25% tensile strength than unprocessed *A. plantensis*	
			↑ moisture-induced plasticization	
Glycerol-pectin-pine-needle	*Chlorella vulgaris*	NA	For 20:50:20:10 blend:	^23^
			↑ Young’s modulus (95.66 MPa)	
			↑ tensile strength	
Starch-glycerol	*Arthrospira platensis*	Microalgae added as powder	3% microalgae achieved:	^24^
			↑ 2.5x tensile strength	
			↑ 1.9x Young’s modulus	
EVA	*Chlorella* sp. HS2	Freeze-drying	10% microalgae content:	^25^
			↑ elongation at break	
			Lipid-free microalgae:	
			↑ ductility and strength	
Styrene-butadiene s	*Chlorella vulgaris* or *Arthrospira platensis*	Powder	↓ overall tensile strength and elongation at break	^26^
			10–20% microalgae content:	
			↑ elongation at break (%) than 5% content	
Nanoclay/bacterial cellulose	*Arthrospira platensis*	Drying, pure or sonicated	3–5 GPa flexural modulus	^19^
			25.5–57 MPa strength	
			Flame-safe	
Carrageenan	*C. vulgaris*	NA	Microalgae collected on the 16th day of cultivation:	^27^
			36.26 MPa tensile strength	
			↑ elongation at break	
			↑ surface	

a Poly­(ethylene-vinyl acetate) (EVA), polylactic acid (PLA), “=” refers to equal or maintained, “↑” refers to increased or improved, “↓” refers to decreased or impaired.

A particular benefit of combining microalgae with biocomposite formulations is the improvement of the mechanical properties of these materials. For example, adding *Chlorella* sp. to poly­(ethylene-vinyl acetate) improved the elongation at break.^22,25^ In contrast, Liao et al. found that the addition of *Arthrospira platensis* to PLA maintained Young’s modulus, whereas the elongation at break, strength and toughness decreased with increasing *A. platensis* content when compared to neat PLA.^22^ Therefore, to obtain effective biocomposites, some key parameters seem to have a direct impact on enhancing biocomposites’ mechanical properties, such as microalgae content used, particle size, cellular component, or harvesting time. Yang et al.^25^ found that lipid-free biomass improved the biocomposites’ mechanical properties. In contrast, Mat Yasin et al.^27^ found that a higher percentage of lipid led to a higher mechanical and thermal stability.

Research into incorporating microalgal and cyanobacterial biomass into paper pulp formulations and papermaking is still in its early stages. Mukherjee et al.^28^ used algal biomass pulp to produce handmade paper. They found that *Rhizoclonium* sp., *Hydrodiction* sp., *Pithophora* sp., and *Cladophora* sp. presented suitable tensile strength (1.12–2.33 kN·m^–1^) due to their high cellulose and hemicellulose content, combined with low lignin and silica levels. *Lyngbya* sp., the only cyanobacterial strain tested, was unsuitable for paper pulp formulation due to its low cellulose and high silica content, leading to a lower tensile strength (0.05 kN·m^–1^). Mukherjee et al.^29^ reported that paper made from pure algal fibers presented poor mechanical strength properties, indicating that the combination of algal pulp with other fibers needed to be exploited to enhance these properties. Furthermore, they found that adding algae to paper formulation enhanced some mechanical properties while impairing others, suggesting that the final application should be considered to this effect. Caprita et al.^30^ found that adding the macroalga *Ulva rigida* to paper formulation improved mechanical properties such as breaking load and length, tearing resistance, folding endurance, and water absorption. Moral et al.^31^ investigated the addition of residual biomass of the macroalga *Ulva* sp. for papermaking, which produced paper sheets with excellent physical properties that exceeded the tear resistance of softwood fibers on their own. Therefore, adding algal biomass into paper formulation could improve the mechanical properties of these materials while reducing the dependence on wood fibers.

Here, the replacement of cellulosic fibers by the native filamentous cyanobacterium (*Leptolyngbya* sp. SB090721) to form new paper-like materials was studied, and the mechanical properties were evaluated. Future research will need to be developed around selecting these parameters, to advance the development of mechanically enhanced biocomposites. This is the first research article to investigate replacing cellulose with these filamentous cyanobacteria in such materials.

## 2 Materials and Methods

### 2.1 Microalgal Strain and Preculture Conditions

Cultures of the isolated *Leptolyngbya* sp. SB090721 ^32^ were grown in 10 L polycarbonate carboys (Nalgene, Thermo Fisher Scientific, Rochester, USA), with a working volume of 9.5 L of BG11 medium at 25 °C. BG11 medium was based on Rippka et al.^33^ medium and prepared following the Culture Collection of Algae and Protozoa (CCAP) protocol.^34^ The cultures were illuminated using warm white LED panels, providing 35 μmol·m^–2^·s^–1^ of light, with a 12 h light/12 h dark photoperiod. Continuous aeration (2 L·min^–1^) was supplied by using filtered (0.2 μm) ambient air from a Blagdon KOY Air 25 aquarium pump (Blagdon, Surrey, UK).

### 2.2 Preparation of Cyanobacterial Biomass Extracts

A quantity of approximately 100 mg of freeze-dried cyanobacterial biomass was weighed out and extracted in 200 mL of a mixture of ultrapure water, methanol, and butanol (15:4:1, v:v:v) at 22 °C for 3 and 24 h as indicated by Teneva et al.^35^ Extracts were pooled and centrifuged at 4,000*g* for 30 min. Then, the solvent was concentrated in a rotary evaporator at 37 °C for 2 h, 0.45 μm filtered, and dried under nitrogen. Extract quantity was determined gravimetrically, and the extract was resuspended in DMSO to give a final concentration of 10,000 μg·mL^–1^. Extracts were tested for sterility by plating 10 μL on tryptic soy agar plates (nutrient-rich and nonselective media).

### 2.3 Biological Assessment of Cyanobacterial Biomass Extracts

#### 2.3.1 Cell Culture

Normal human dermal fibroblasts (Neo-NHDF, PromoCell, USA) were cultured in DMEM low glucose (Gibco, USA) supplemented with 10% fetal bovine serum (Sigma, UK), 1% penicillin/streptomycin (P/S) (Sigma, UK), and 1% l-glutamine (Sigma, UK). Cells were seeded at a density of 5000 cells·cm^–2^ and kept in a humidified incubator at 37 °C, with 5% CO_2_. Culture media was replaced every 2 days, and upon reaching 80% confluency, cells were detached using trypsin/EDTA (0.25% w/v trypsin/0.02% EDTA, Gibco) and split on a 1:2 regime. At passage 11 cells were seeded in 48-well plates, at a seeding density of 1500 cells/well, and cultured for 7 days with the collected extracts prepared in section 2.2, diluted in supplemented DMEM to the concentrations of 1, 10, and 100 μg·mL^–1^. Cell culture in standard culture media was used as a control. Samples were collected after 1, 3, and 7 days of culture for analysis.

#### 2.3.2 Metabolic Activity

The cells’ metabolic activity was measured with the 3-dimethylthiazol-2,5-diphenyltetrazolium bromide colorimetric assay (MTT), prepared according to the manufacturer’s instructions (Sigma-Aldrich, Dorset, UK). The supernatant collected from each well was transferred to a 96-well plate and read using a spectrophotometer (FLUOstar Omega Microplate Reader, BMG Labtech) at 570 nm. The test was performed in triplicate, and the results were treated with GraphPad Prism 10 software (GraphPad Software, USA).

#### 2.3.3 Live/Dead Assay

Cytotoxicity was assessed with a double staining live/dead kit (Thermo Fisher, UK), prepared according to the manufacturer’s instructions, leading to two stock solutions with a final concentration of 4 μM ethidium homodimer-1 (EthD1) and 2 μM calcein. The culture media was removed from the wells, and cells were washed twice with Dulbecco’s Phosphate Buffer Solution (DPBS). The stock solutions were added to the wells, and the plates were incubated for 30 min at room temperature and protected from light. Stained cells were observed directly in the well using a fluorescence microscope (EVOS M5000, Thermo Fisher, UK) equipped with 10x and 20x objectives. Live cells were visualized using the FITC (green) and RFP (red) filter, to depict the live and dead cells, respectively.

#### 2.3.4 Cell Morphology

The morphology of the fibroblasts was observed via staining of the cell’s cytoskeleton and nucleus on days 1 and 7 only. At each time point, samples were washed with DPBS and fixed in 4% paraformaldehyde (PFA) (v/v; Thermo Scientific, UK) for 15 min at room temperature. Samples were washed 3 times in DPBS, and permeabilized with a solution of 0.1% Tween20/PBS (v/v) for 5 min. A solution containing phalloidin-tetramethylrhodamine B isothiocyanate peptide (Sigma-Aldrich) diluted in DPBS/0.1%Tween 20 (1:1000 ratio) was applied to each well for 30 min at room temperature, protected from light. Samples were washed 3 times with DPBS and subsequently incubated in a 4′,6-diamidino-2-phenylindole (DAPI; Sigma-Aldrich) solution, created by dissolving DAPI in DPBS (1:2500), and incubated for 1 h at room temperature, light-protected. Finally, samples were washed with DPBS once, and stained cells were observed directly in the well using a fluorescence microscope (EVOS M5000, Thermo Fisher, UK) equipped with 10x and 20x objectives. Live cells were visualized using the DAPI (Blue) and RFP (red) filter, to depict the cell nuclei and cytoskeleton, respectively.

### 2.4 Biocomposite Production

Biocomposites were prepared as described by Ekins-Coward et al.^36^ Briefly, a 1% (w/v) chitosan stock solution and a 0.3% solid concentration stock solution of Georgia Pacific hardwood (HW), softwood (SW), and Cellulose Lab microfibrillated cellulose (MFC) in BG-11 were prepared and autoclaved at 121 °C for 25 min. The required volume of cyanobacterial culture for each testing condition was collected by centrifugation at 4,000*g* for 10 min and concentrated in 20 mL of BG-11. This concentrated culture was then slowly added to a suspension containing a mixture of chitosan and pulp, while stirring slowly. The suspension was poured onto prewetted and leveled Whatman grade 41 filter paper (pore size 20 μm, GE Healthcare, Chicago, USA) in a funnel under a vacuum. The resulting biofilm was removed from the filter, pressed to remove excess water, peeled off, and placed on a drying ring to prevent shrinkage during overnight drying.

### 2.5 Physicochemical Characterization

#### 2.5.1 Fourier Transform Infrared Spectroscopy

FTIR spectra were recorded using a Cary 630 FTIR Spectrometer (Agilent Technologies, Santa Clara, USA) to analyze the functional groups present in the biocomposites. The samples were scanned in the range of 4000–650 cm^–1^ using a resolution of 2 cm^–1^. All the obtained spectra resulted from an average of scans of three independent biological replicates (*n* = 3).

#### 2.5.2 Mechanical Properties

The tensile properties of the biocomposites were measured using a universal testing machine (Shimadzu, Japan) following the British Standard ISO 1924-3:2005 for Paper and Board Determination of Tensile Properties, with some modifications.^37^ Specimens were cut using an in-house dog-bone-shaped cast and a scalpel. The dimensions of the specimens were as follows: overall length, 70 mm; gauge length, 35 mm; thickness, 0.3–0.4 mm; and width, 9.27 mm. The specimens were clamped onto the machine, and the rate of elongation was set to 1 mm·min^–1^. Each experimental condition was tested in nine independent replicates (*n* = 9). From each biocomposite, three specimens were cut in a dog bone shape with the following dimensions: Tensile strength (σ_b_^T^), strain at break (ε_T_), tensile energy absorption (*W* ^b^_T_), tensile stiffness (*E* ^b^), and elasticity modulus (*E*) were calculated according to BS ISO 1924-3:2005.

#### 2.5.3 Optical Microscopy and Scanning Electron Microscopy

Biocomposites were observed in duplicates under the Mitutoyo Quick Scope before and after the tensile test with magnifications ranging from 0.25x to 3x. Images include a bar scale obtained with an external calibrator and added with ImageJ. Biocomposite specimens were dehydrated with carbon dioxide in a Baltec Critical Point Dryer (Leica Geosystems Ltd., Milton Keynes, UK). Specimens were mounted on an aluminum stub with Acheson’s SilverDag (Agar Scientific, Stansted, UK) and dried overnight. Samples were then coated with gold particles of 5–10 nm size using a PolaronSEM Coating Unit (Quorum Technologies Ltd., Laughton, UK) and imaged using a Tescan Vega LMU Scanning Electron Microscope with Tescan supplied software (Tescan, Girton, UK).

### 2.6 Effect of Cellulose Replacement by Cyanobacterial Biomass

The effect of replacing cellulose fibers with locally isolated cyanobacterial biomass on the mechanical properties of biocomposites was evaluated. Biocomposites were produced as described in Section 2.3 with varying percentages of hardwood (HW) and softwood (SW), the two main cellulosic components. Three conditions were studied: 0%, 3%, and 30% replacement, each tested in nine replicates (*n* = 9). Table 2 shows the final composition of each component in the final biocomposite.

**Table 2:** Biocomposite Composition in Percentages (w/w) for Each Component^a^

**Component** % (w/w)	**Control (0%)**	**Standard (3%)**	**High Biomass (30%)**
**HW**	41.5	40.0	26.5
**SW**	41.5	40.0	26.5
**MFC**	7.00	7.00	7.00
**Chitosan**	10.0	10.0	10.0
**Cyanobacteria**	0.00	3.0	30.0

a Biocomposites were prepared as described by Ekins-Coward^36^ with increasing concentrations of cyanobacterial biomass. HW = Georgia Pacific hardwood, SW = softwood, MFC = Cellulose Lab microfibrillated cellulose.

### 2.7 Statistical Analysis

A one-way ANOVA analysis was carried out in GraphPad Prism 10 using a *p*-value of 0.05. Results were expressed as the average value ± the standard deviation of at least three independent biological replicates.

## 3 Results and Discussion

### 3.1 Cytotoxicity of *Leptolyngbya* SB070921 sp. Extracts

The cytotoxic effect of three extracts (100, 10, 1 μg·mL^–1^) of *Leptolyngbya* SB070921 sp. on human dermal fibroblasts (Neo-NHDF) was evaluated by studying the cells’ metabolic activity, viability, and morphology. The MTT assay results (Figure 1a) show a sustained metabolic activity of these cells when exposed to the extracts with low concentrations (1 and 10 μg·mL^–1^), comparable to that of the control. For the highest concentration, 100 μg·mL^–1^, the metabolic activity of the cells was significantly lower at all time points, being close to 0 by day 7, implying cell death. Besides, cell viability via live/dead (Figure 1b) showed a similar trend in which there was an evident reduction of living cells on days 3 and 7, coupled with the loss of the typical spindle shape of healthy fibroblast cells (Figure 1c).^38^ No significant cytotoxic effect was observed for 10 and 1 μg·mL^–1^ extracts when compared to the control sample. Both extracts also showed an increasing cell concentration of fibroblasts on day 7, forming a dense tissue similar to that produced by the control sample (Figure 1b,c). The genus *Leptolyngbya* is well-known for its rich and diverse array of bioactive metabolites, with cytotoxic activities, including antimicrobial, antiproliferative, antiviral, antifungal or antiprotozoal.^39^ Gara-Ali et al.^40^ found that lipophilic extracts of their isolated *Leptolyngbya* sp. showed antiproliferative activity (IC_50_ 0.86 mg·mL^–1^) against colon cancer cell lines. The antiproliferative activity was attributed to compounds such as monoterpenes (α- and β-pinene and limonene), diterpenes (phytol), and γ-sitosterol. Furthermore, Cui et al.^41^ isolated leptolyngbyolides from *Leptolyngbya* sp. which exhibited cytotoxicity against cancer cell lines and actin-depolymerizing activity. Other antiproliferative compounds found in the *Leptolyngbya* genus include Coibamide A, Dolastatin 12, Palmyrolide A or Leptazoline B.^39^ In contrast, 100 μg·mL^–1^ extracts of *Leptolyngbya* sp. KIOST-1 did not show a significant cytotoxic effect on Vero cell lines using the lactate dehydrogenase assay.^42^ *Leptolyngbya* sp. KIOST-1 was also assessed for oral acute toxicity and genotoxicity, showing no toxic effect.^43^ Nevertheless, the development of dried-formulation paper biocomposites, which include unprocessed biomass, will require further investigation to determine the safety of these materials according to their final application. Incorporating cyanobacterial biomass could also provide additional properties (antifungal, antimicrobial, antiviral or antiprotozoal) to these novel materials.^44^

**Figure 1:**
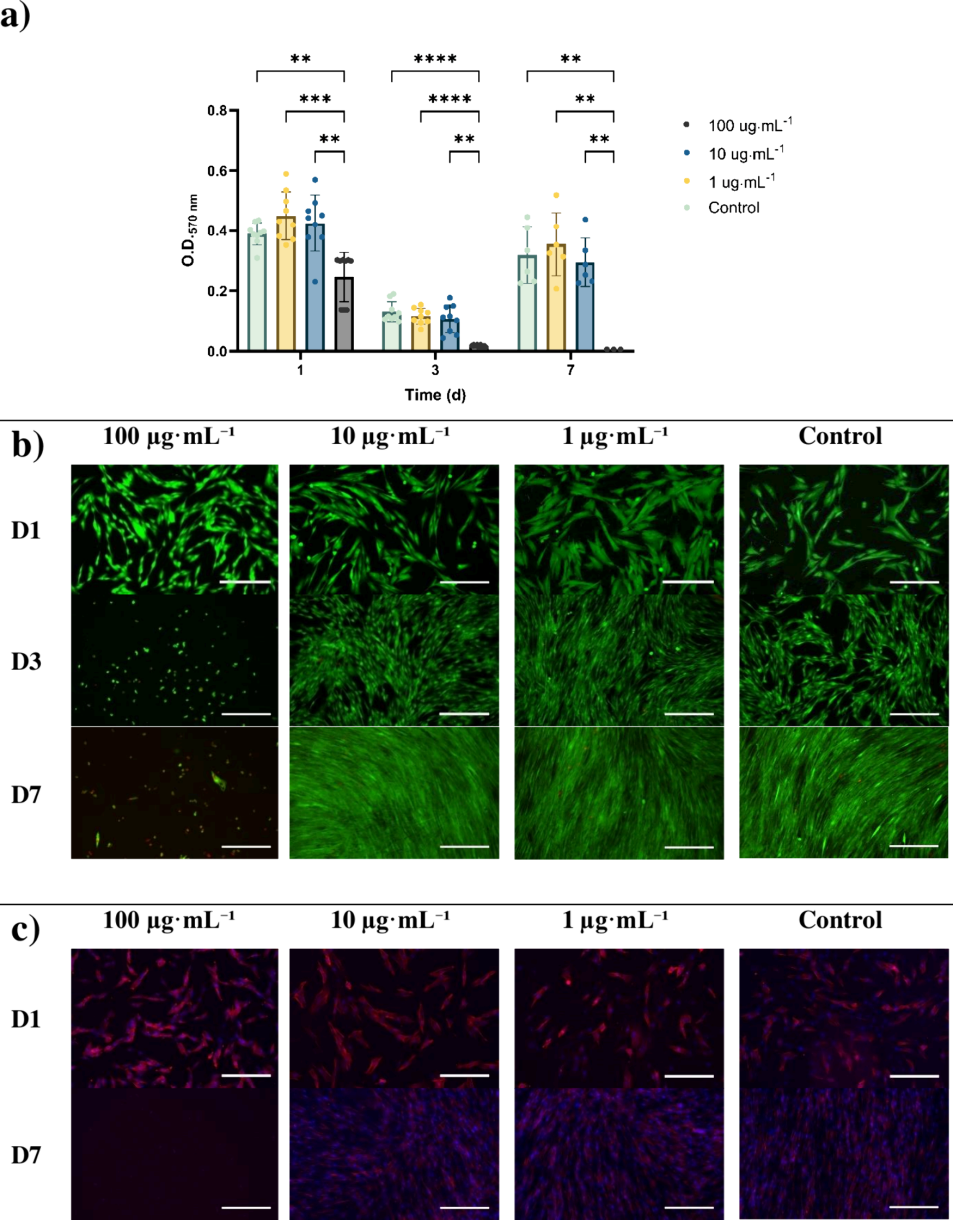
**Proliferation of cells cultivated with *Leptolyngbya* sp. SB090721 extracts (100, 10, 1, and 0 μg·mL** ^**–1**^ **) via MTT assay (*n* = 3), cell viability using live/dead and cell morphology.** a) Metabolic activity using MTT absorbance values. Results presented as mean ± s.d. *p < 0.05, **p < 0.01, ****p < 0.0001. b) Cell attachment and viability on days 1, 3, and 7 (living cells in green and dead cells in red). c) Confocal images of fibroblasts using DAPI staining for the nucleus (in blue), phalloidin for the cell body (in red) and vinculin for actin filaments (in green). All scale bars are equal to 300 μm.

### 3.2 Biocomposite Production and Characterization

This is the first report of using the filamentous cyanobacterium *Leptolyngbya* sp. SB090721 to replace cellulosic fibers in paper-like biocomposites. The three biocomposite formulations produced and their characterization can be observed in Figure 2. Increasing cyanobacterial biomass (0%, 3%, 30%) on biocomposites can be observed, as the color changes from white to light and dark green. The FTIR analysis of the three biocomposites (Figure 2a–c, ii) showed differences in the functional groups attributed to the increasing cyanobacterial biomass content.

**Figure 2:**
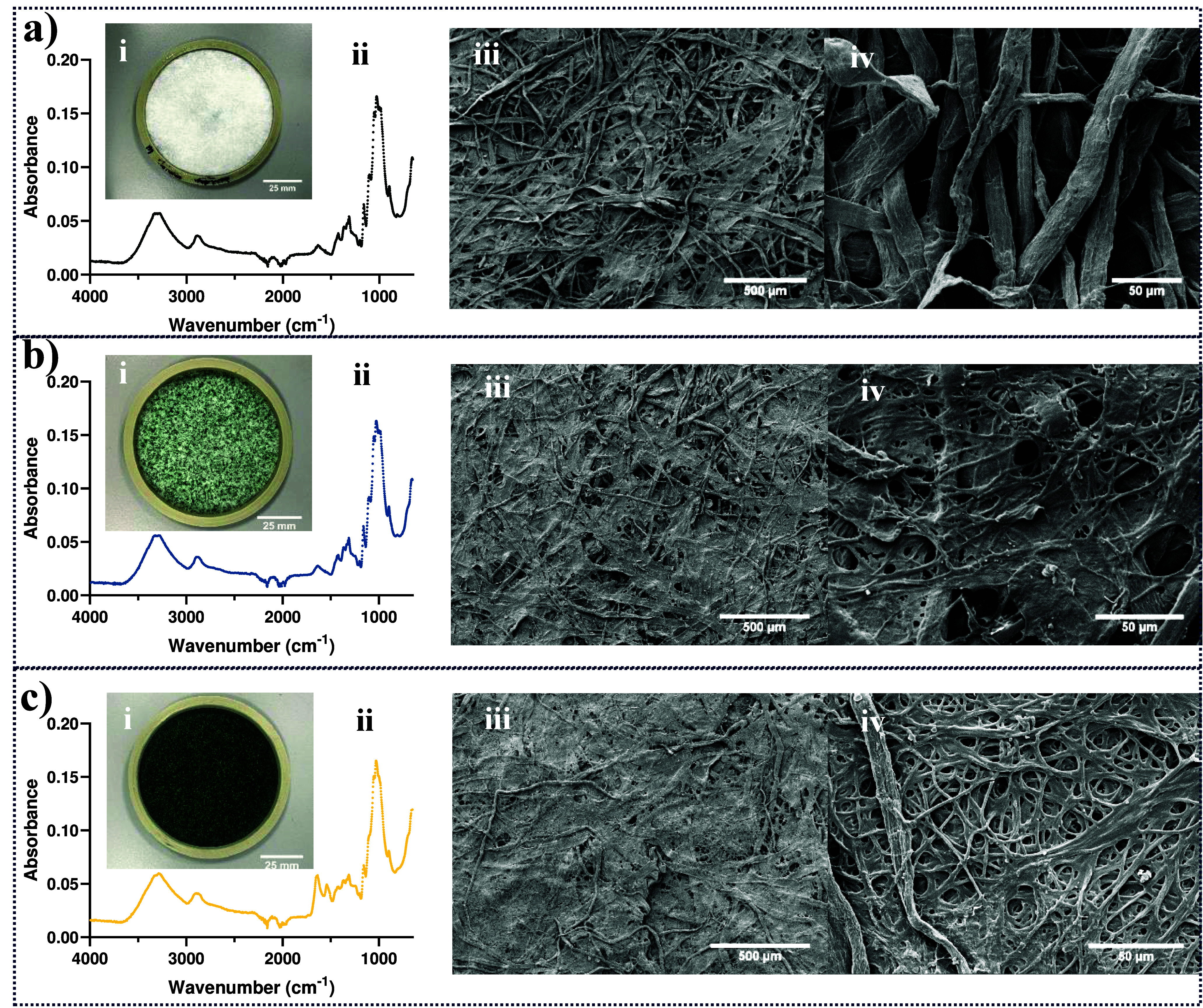
**Production of biocomposites with *Leptolyngbya* sp. SB090721 biomass.** a) Control biocomposite, b) standard biocomposite (3% cellulose replacement), c) high biomass biocomposite (30% cellulose replacement). (i) Images of the final biocomposite in 3D-printed cast; (ii) characteristic FTIR spectra of each biocomposite; (iii) SEM micrograph overview of biocomposites (scale bar equal to 500 μm); (iv) SEM micrograph close-up showing the differences in the fiber structure of each biocomposite (scale bar equal to 50 μm).

Absorption bands were present in all samples at two wavenumber regions: 3500–2800 and 1650–650 cm^–1^. These bands are characteristic of cellulose, hemicellulose, and lignin, the main components of softwood and hardwood.^45^ The three biocomposite formulations shared absorption bands in the region 3291–3350 cm^–1^ corresponding to O—H stretching, as well as the intramolecular hydrogen bonds in polysaccharides.^46^ The band at 2894 cm^–1^ was attributed to the CH stretching vibration of hydrocarbon constituents in polysaccharides.^47^ The presence of chitosan could be inferred by the presence of N-acetyl groups, which exhibit a weak band at around 1650 cm^–1^ (C=O stretching of amide I) and 1321 cm^–1^ (C—N stretching of amide III).^48,49^ However, other typical bands at 1550 cm^–1^ (to N—H bending of amide II) and 1589 cm^–1^ (to N—H bending of amine I) were not found, which was probably due to overlapping peaks. Sharp bands were identified on the region of 890–1150 cm^–1^ which were assigned to stretching vibrations of O—H, C—H and —O— groups and to the β-glycosidic bond between glucose units in cellulose.^47,48^ Nevertheless, high biomass composites showed two distinct bands at 1650 and 1530 cm^–1^. The 1650 cm^–1^ band (C=O stretching of amide I) of the 30% loaded sample exhibited twice the intensity of the control and standard biocomposite bands, reflecting the high protein content of the cyanobacteria. The 1530 cm^–1^ band (bending vibration of C—N and stretching vibration of N—H of amide II) also distinctly appeared on the high biomass composite,^50^ for the same reason. *Leptolyngbya* sp. SB090721 biomass is 31.3% protein, as described by Serrano-Blanco et al.^32^

SEM micrographs (Figure 2iii and Figure 2iv) revealed the structural differences between the biocomposite formulations. The control biocomposite showed a mix of different cellulosic fiber sizes, producing a tight fiber network with high porosity similar to that produced by Ekins-Coward.^51^ Micrographs at higher magnification showed these neat fibers intertwined among others. Standard biocomposites with a 3% cellulose replacement showed a compact structure with an apparent lower porosity due to the inclusion of the cyanobacterial biomass. Cyanobacterial filaments were found to connect cellulosic fibers, creating a thinner network between fibers with random areas fully covered. The high biomass biocomposite with a 30% cellulose replacement showed a completely different structure with fewer cellulosic fibers and a structure dominated by an entangled mesh of smooth cyanobacterial filaments. This microscopic structure was dominated by the cyanobacterial filaments with a fiber width of ∼1.5 μm, whereas the softwood and hardwood fibers with a fiber width of ∼21 μm were less abundant.

### 3.3 Effect of Cellulose Replacement by Cyanobacterial Biomass on Tensile Properties

Here, repurposing entrapped microalgal biocomposites into new paper-like materials was evaluated to assess the replacement of cellulose fibers with nonwood renewable fibers. The mechanical response of biocomposites containing 0%, 3% and 30% cellulose replaced with filamentous cyanobacterial biomass is presented in Figure 3 and Figure S1.

**Figure 3:**
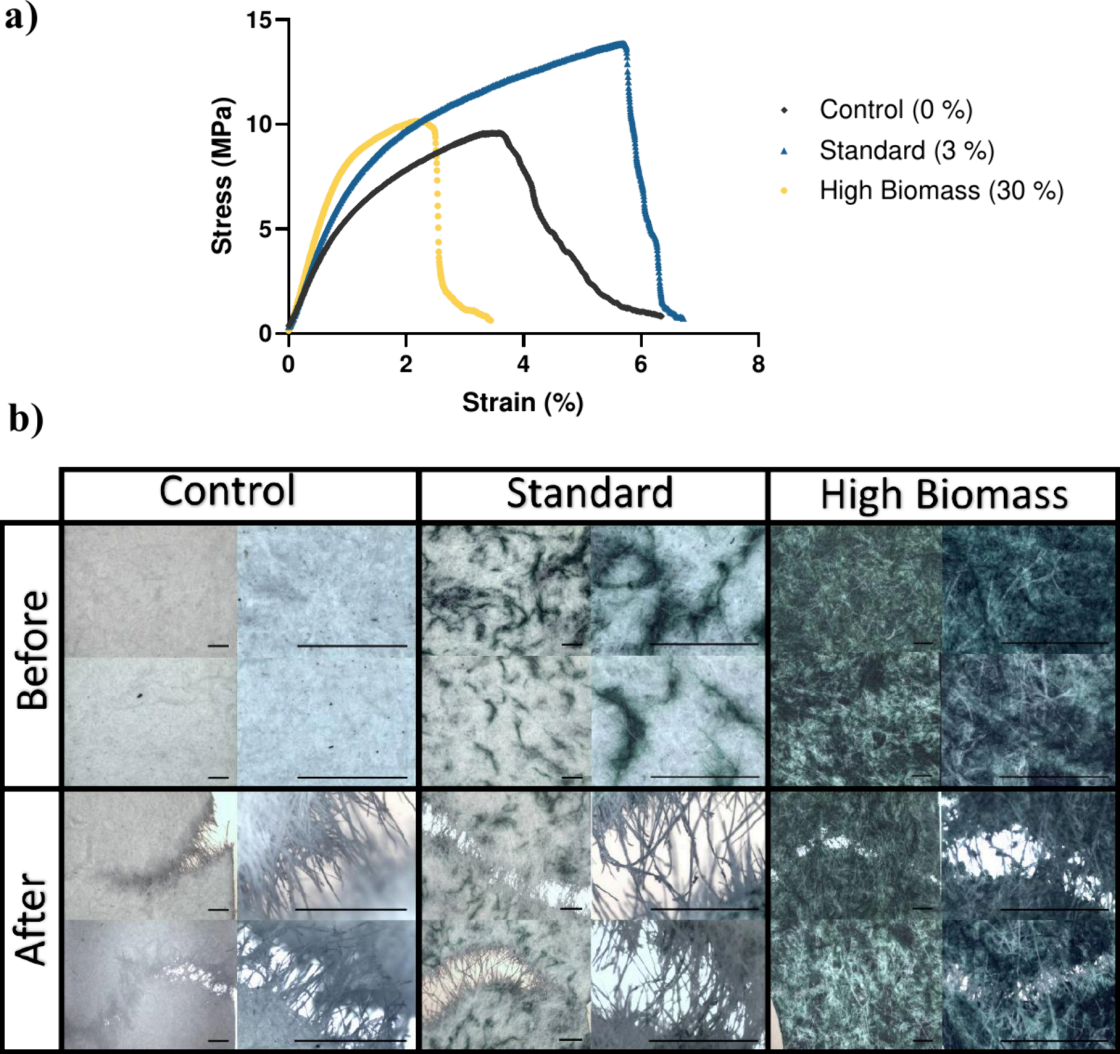
**Tensile testing of biocomposites prepared at different concentrations of cyanobacterial biomass (0%, 3%, 30%).** a) Typical elongation curve showing the different behavior of each specimen according to their composition. b) Microscopic images showing the specimens before and after rupture in duplicates. The tree formulations showed a compact structure before the test with details of the cyanobacterial filaments when present and separation of cellulosic fibers after rupture. The scale bar is equal to 100 μm.

The elongation curve showed clear differences in the tensile behavior of these biocomposites. The replacement of 3% cellulose with cyanobacteria improved the elastic and plastic properties of the standard biocomposite when compared to the control biocomposite (0%). The addition of 30% cyanobacterial biomass also improved the elastic modulus (Young’s modulus) of the material. Still, it impaired its plastic behavior, resulting in the characteristic curve of brittle materials breaking when elastic deformability was exceeded. The control biocomposite exhibited moderate elastic properties, whereas its plastic behavior fell between the standard (3%) and high biomass composites (30%). Figure 3b shows the microscopic appearance of the biocomposites before and after the rupture test. The high biomass biocomposite showed a more compact structure, with fewer cellulosic fibers (Figure 2c, iv). Therefore, it seems that a 30% replacement of cellulosic fibers with cyanobacteria microfilaments impaired the ability of the material to transfer shear stress, thereby reducing the plastic behavior of the material. A similar behavior has been observed on corn starch biocomposites containing 20% of *Spirulina* or *Scenedesmus*, which did not show plastic deformation and broke when the yield point was exceeded.^52^ The integrity of the cell wall during biocomposite fabrication may play a significant role in this effect.

Replacing cellulose fibers with filamentous cyanobacterium significantly affected the tensile properties of paper-like biocomposites (Figure 4). The addition of cyanobacterial biomass at 3% and 30% improved or maintained most of the tensile properties studied.

**Figure 4:**
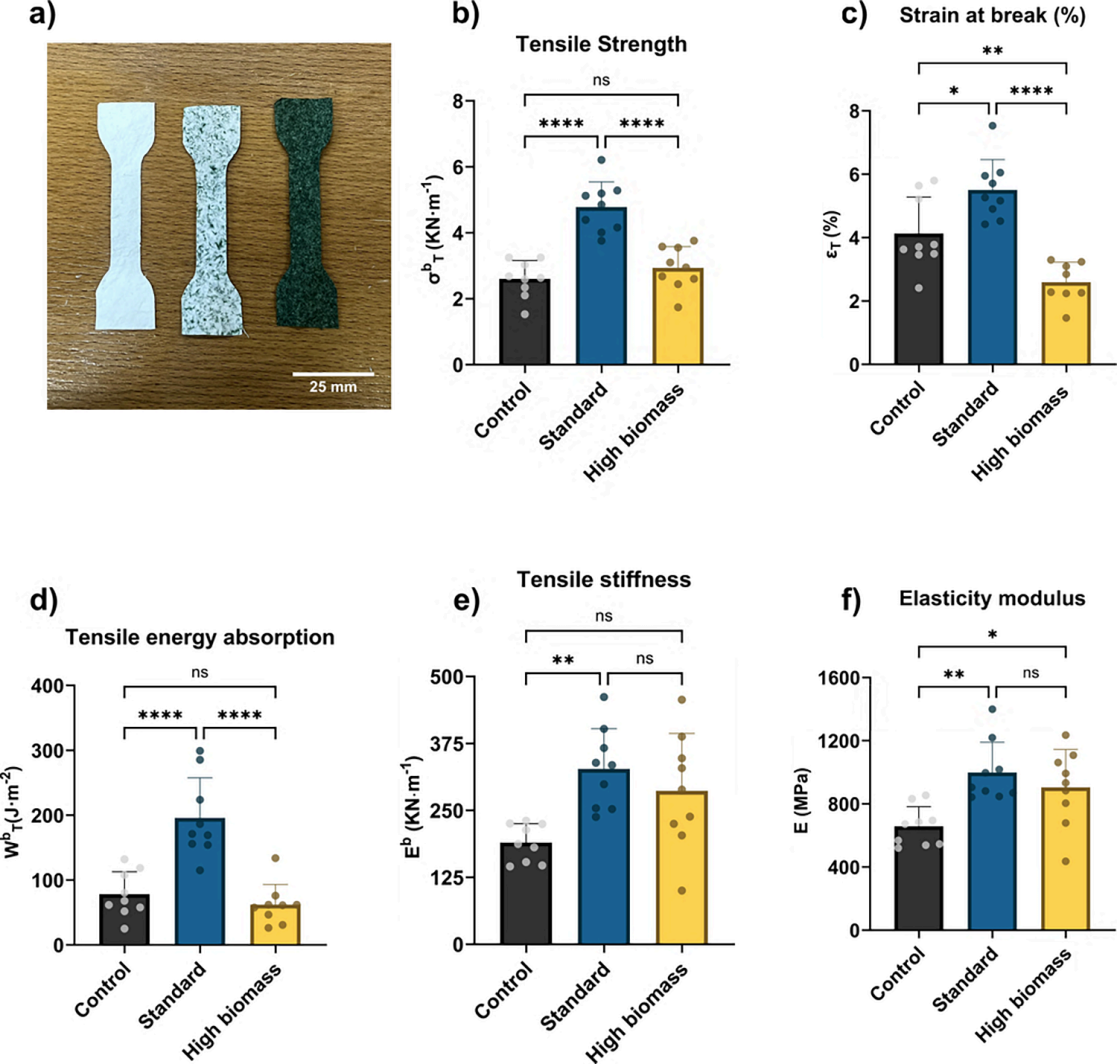
**Effect of cellulose replacement by cyanobacterial biomass on tensile properties.** a) Image showing dog-bone test pieces required for tensile testing sliced from each biocomposite. b–f) Comparison of the characteristic paper and board tensile properties for each specimen (control 0% in black, standard with 3% replacement in blue, and high biomass with 30% replacement in yellow). b) Tensile strength, c) strain at break (%), d) tensile energy absorption, e) tensile stiffness, and f) elasticity modulus. Data represent the mean of nine independent biological replicates (*n* = 9) ± s.d. *p < 0.05, **p < 0.01, ****p < 0.0001, ns, not significant or *p* > 0.5.

Standard treatment achieved a significantly (*p* < 0.001) higher tensile strength (4.8 kN·m^–1^) when compared to the control. The 30% cellulose replacement (high biomass biocomposite) maintained the tensile strength of the biocomposite when compared to the control. As expected from the stress–strain curve (Figure 3a), the high biomass biocomposite showed a lower strain at break (2.6 ± 0.6%), typical of a brittle material, whereas the control (4.1 ± 1.2%) and the standard (5.5 ± 1.0%) achieved higher strains before break and were able to undergo higher plastic deformation. As observed on the high biomass SEM micrographs, the content and orientation of the cellulosic fibers differ from the standard and control. Indeed, fiber content and orientation have a significant effect on the mechanical properties of biocomposites.^53^ The standard biocomposite displayed significantly (*p* < 0.0001) higher tensile energy absorption (195.6 ± 61.99 J·m^–2^), which is an important property for the durability of materials. The standard biocomposite also showed a higher tensile stiffness (327.4 ± 75.14 kN·m^–1^) than the control and high biomass treatments. Finally, both the control and standard showed enhanced elasticity modulus (997.1 ± 192.5 and 903.4 ± 240.8 MPa, respectively) compared to the control (656.5 ± 125.1 MPa). Altogether, these properties indicated that the standard biocomposite showed improved elastic and plastic capabilities when compared to the control, whereas the high biomass composite improved the elastic properties while maintaining its plastic behavior.

Table 3 shows the comparison of the tensile strength and modulus of elasticity of the biocomposite hereby prepared and those reported in the literature. *Leptolyngbya* sp. SB090721 biocomposites showed comparable tensile strength to other biocomposites made of starch and 20% of *Spirulina*, *Scenedesmus* or *Nannochloropsis.* ^52^ Biomaterials containing plastic polymers like polylactic acid with *Arthrospira platensis* or carrageenan with *Chlorella vulgaris* showed superior tensile properties to the ones prepared in this study.^22,27^ These differences can be attributed to the inherent tensile properties of their base components (PLA or carrageenan) rather than to the addition of microalgae into their formulation. *Leptolyngbya* sp. SB090721 biocomposites showed tensile properties similar to those of paper and paperboard materials.

**Table 3:** Tensile Strength and Elasticity Modulus Values for Biocomposites Incorporating Microalgae in Their Formulation

**Biomaterial**	**Tensile Strength (MPa)**	**Elasticity Modulus (MPa)**	**Reference**
Cellulose*-Leptolyngbya* sp. SB090721 biocomposite	9.48–14.6	903–997	This study
*Lyngbya* fibers	215	24000	^54^
Corn starch and *Spirulina*	13.5	1276	^52^
Corn starch and *Scenedesmus*	10.8	1257	
Corn starch and *Nannochloropsis*	11. 7	800	
Residual microalgal biomass	18.7–33.3	583–780	^18^
Starch-glycerol and *Arthrospira platensis*	2.61–6.51	83–158	^24^
PLA and *Arthrospira platensis*	20–55	2001–2599	^22^
Styrene-butadiene with *Chlorella vulgaris* or *Arthrospira platensis*	4.41–5.4	NA	^26^
Carrageenan and *C. vulgaris*	36.26	NA	^27^
Paper and paperboard	5–45	0.5–20	^51^

Biocomposites utilizing unprocessed microalgae may benefit from strains that contain a higher content of cellulose or carbohydrates as found by Mukherjee et al.^28^ The cyanobacterial cell envelope comprises a sheath, membrane, and a thick layer of membrane.^55^ However, microalgae contain rigid cell wall (species-dependent) comprising (1) an external cell wall with a polysaccharide matrix (including pectin, agar, alginate, and algaenan) and (2) an internal cell wall (containing hemicellulose, pectin, and glycoproteins within a microfibrillar cellulosic matrix).^56^ Therefore, unprocessed filamentous microalgae, excluding cyanobacteria, could enhance mechanical properties due to their reinforced cell wall.

Unlike some of the biocomposites in Table 3, which include complex machinery and require energy-intensive pretreatments, these biocomposites were produced at room temperature by using unprocessed cyanobacterial biomass without any energy-intensive processing. In contrast, although macroalgae are easier to harvest and can significantly improve the tensile strength of paper, they require cellulose extraction through drying and a chemical treatment or must be ground into a fine powder before being incorporated into the pulp.^30,31,57^ In here, the replacement of cellulosic fibers with untreated cyanobacterial biomass improved or maintained the tensile properties, suggesting that nonwood fibers can be used in place of cellulosic fibers to reduce environmental impact. Coupling the growth of *Leptolyngbya* sp. SB090721 to a wastewater treatment facility could provide additional advantages by reducing the cost of the biomass production.

## 4 Conclusions

Novel cellulosic biocomposites were developed, replacing wood-based cellulose fibers with *Leptolyngbya* sp. SB090721 biomass: control 0%, standard 3% and high biomass 30% (w/w). The cytotoxicity of the extracts exhibited cytocompatibility below 100 ug·mL^–1^, unveiling potential applications of biocomposites. FTIR characterization of high biomass biocomposites showed two characteristic bands at 1650 and 1530 cm^–1^, indicating a high protein content of the cyanobacterium. The high biomass specimen displayed a distinct structure with a finer mesh, which was attributed to its elevated cyanobacterial content. The addition of cyanobacteria biomass into biocomposite formulations in place of cellulose maintained or enhanced tensile properties and elasticity at lower loadings. Overall, these results show that, in terms of biocomposite strength, cyanobacterial biomass can replace cellulosic fibers in paper/paperboard materials. It was demonstrated that unprocessed cyanobacterial biomass, without requiring energy-intensive pretreatments, can partially replace wood fibers and support the transition toward a more sustainable industry. Future studies should explore how strains with more rigid cell walls and membranes influence the substitution of cellulose in biocomposites and their tensile properties.

## Supplementary Material



## Notes

The elongation curves for each specimen tested and the estimated indices for each specimen (PDF)
