# Supplementary material for: Mechanical Reinforcement of Paper Biocomposites Using Filamentous Cyanobacteria
Source: ACS Sustain Chem Eng. 2025 Oct 1;13(41):17083–93. doi: 10.1021/acssuschemeng.5c02889 (PMC12541801; doi:10.1021/acssuschemeng.5c02889)
Supplement: Supplementary file 1 [file sc5c02889_si_001.pdf]

Supplementary Information

Mechanical Reinforcement of Paper Biocomposites Using Filamentous Cyanobacteria

Sergio Serrano-Blanco[a], Priscila Melo[a], Adam P Harvey[a], Sharon Velasquez-Orta[a,]*

[a] School of Engineering, Merz Court, Newcastle University, Newcastle upon Tyne, NE1 7RU, UK

*Corresponding author:

Name: Sharon Velasquez-Orta

Address: School of Engineering, Merz Court, Newcastle University, Newcastle upon Tyne NE1 7RU, United Kingdom

Email: sharon.velasquez-orta@newcastle.ac.uk

This supporting information consists of 2 pages and includes two figures: the elongation curves for each specimen tested and the estimated indexes for each specimen.

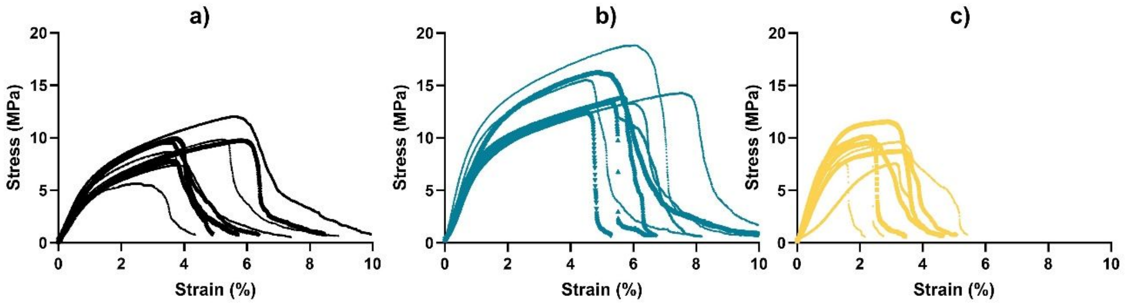

Figure S1. Elongation curves for each specimen tested (n=9). a) control, 0 % replacement; b) standard, 3 % replacement; c) high biomass, 30 % replacement.

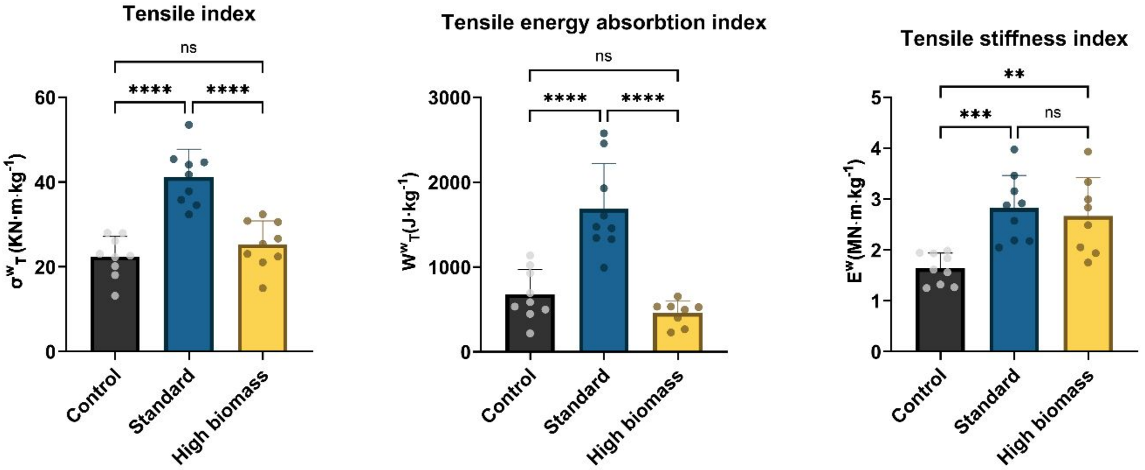

Figure S2. Estimated indexes for each specimen tested (n=9). From left to right, tensile index, tensile energy absorption index and tensile stiffness index. Note that these values have been estimated assuming that the weight of each biocomposite remained constant, with the cellulose replaced by an equal amount of cyanobacteria.
